# Prediction of Soil Properties Using Vis-NIR Spectroscopy Combined with Machine Learning: A Review

**DOI:** 10.3390/s25165045

**Published:** 2025-08-14

**Authors:** Su Kyeong Shin, Seung Jun Lee, Jin Hee Park

**Affiliations:** Department of Environmental and Biological Chemistry, Chungbuk National University, Cheongju 28644, Chungbuk, Republic of Korea; ztnrudz12@naver.com (S.K.S.); jjun3607@naver.com (S.J.L.)

**Keywords:** Vis-NIR spectroscopy, machine learning, soil nutrients, real-time measurement, data preprocessing, precision agriculture

## Abstract

Stable crop yields require an appropriate supply of essential soil nutrients such as nitrogen (N), phosphorus (P), and potassium (K) based on the accurate diagnosis of soil nutrient status. Traditional laboratory analysis of soil nutrients is often complicated and time-consuming and does not provide real-time nutrient status. Visible–near-infrared (Vis-NIR) spectroscopy has emerged as a non-destructive and rapid method for estimating soil nutrient levels. Vis-NIR spectra reflect sample characteristics as the peak intensities; however, they are often affected by various artifacts and complex variables. Since Vis-NIR spectroscopy does not directly measure nutrient levels in soil, improving estimation accuracy is essential. For spectral preprocessing, the most important aspect is to develop an appropriate preprocessing strategy based on the characteristics of the data and identify artifacts such as noise, baseline drift, and scatter in the spectral data. Machine learning-based modeling techniques such as partial least-squares regression (PLSR) and support vector machine regression (SVMR) enhance estimation accuracy by capturing complex patterns of spectral data. Therefore, this review focuses on the use of Vis-NIR spectroscopy for evaluating soil properties including soil water content, organic carbon (C), and nutrients and explores its potential for real-time field application through spectral preprocessing and machine learning algorithms. Vis-NIR spectroscopy combined with machine learning is expected to enable more efficient and site-specific nutrient management, thereby contributing to sustainable agricultural practices.

## 1. Introduction

Since soil is a major source of essential nutrients for crop growth, characterizing soil properties is important for effective nutrient management [1]. Soil fertility is affected by soil physicochemical properties, including soil parent material, pH, cation exchange capacity (CEC), base saturation, and organic matter content [2]. Among the essential nutrients, N, P, and K are particularly important sources of crop nutrition for protein synthesis, plant development, energy transfer, root growth, photosynthesis, and physiological functions [3]. Supplying appropriate amounts of N, P, and K fertilizers contributes to proper crop growth, development, and physiological functions by ensuring sufficient nutrient availability in the soil [4]. Therefore, to ensure proper nutrient management, it is necessary to precisely determine the nutrients present in the soil to prevent both deficiencies and excesses. Laboratory methods for determining soil nutrient contents provide accurate measurements from randomly collected soil samples; however, these methods are often complex and time-consuming in sampling and sample preparation, and they do not provide real-time information on nutrient status in the field [5,6].

Visible (Vis)–near-infrared (NIR) reflectance spectroscopy has been widely used to estimate soil properties, including soil texture, pH, organic matter, inorganic C, macronutrients, and micronutrients [7,8,9]. Reflectance spectroscopy measures light reflected or scattered from solids, liquids, or gases as a function of wavelength. Most soil components show fundamental vibrations in the mid-infrared (MIR) region (2500–25,000 nm), while their overtones and combinations are observed in the NIR region (400–2500 nm) [10]. Spectral reflectance methods are more rapid, non-destructive, and cost-effective, requiring no sample preparation and avoiding the use of chemicals [11]. Spectra signatures of different materials are distinguished based on their reflectance and absorbance characteristics [10]. The Vis-NIR spectra of soils provide both physical and chemical information including organic and inorganic materials present in the soil [1,12]. Since soil properties exhibit unique spectral signatures, soil nutrients can be predicted by selecting appropriate wavelength regions. For example, Zhou et al. [13] detected the on-the-go total nitrogen (TN) concentration using absorbance at 1070, 1130, 1245, 1375, 1550, and 1680 nm. An et al. [14] also developed a potable TN detector using NIR spectroscopy with absorbance data at 940, 1050, 1100, 1200, 1300, and 1550 nm.

To predict soil nutrients through Vis-NIR spectroscopy, a calibration model that integrates spectral data with measured soil parameters is required [15]. Chemometric modeling techniques can handle numerous soil variables including complex spectral patterns [7]. Partial least-squares regression (PLSR), one of the methods most frequently used to develop calibration models, has been widely used to estimate TN content [16,17]. However, since the relationship between TN and spectral data is rarely linear, prediction using a linear model is limited in accuracy [18].

Machine learning algorithms can accommodate nonlinear relationships between spectral data and soil parameters [19]. For example, support vector machine regression (SVMR), artificial neural networks (ANNs), and the extreme learning machine (ELM) can model complex nonlinear relationships and have shown higher accuracies compared to linear methods [13,17,20]. Zhang et al. [21] reported that SVMR (validation coefficient R^2^ = 0.810, residual predictive deviation (RPD) = 2.129) showed great performances in predicting soil TN content compared to PLSR (validation R^2^ = 0.634, RPD = 1.838).

Although the use of Vis-NIR spectroscopy combined with machine learning has rapidly advanced in nutrient prediction, research on its application to on-site agricultural soils is limited. While laboratory-based results of Vis-NIR spectroscopy for soil nutrient prediction are useful, on-site measurement of soil is more important for precision agriculture. Zhou et al. [22] reported that a developed soil TN and moisture detector based on NIR spectroscopy showed validation coefficients of 7832 and 8882 for TN and moisture content, respectively. Mouazen and Kuang [23] found that an online Vis-NIR spectroscopy sensor showed acceptable accuracy in predicting P with a coefficient of determination (R^2^) and an RPD of 0.60 and 1.5, respectively. These field-applicable detectors not only perform real-time and non-destructive diagnosis but also provide decision support for variable-rate fertilization in precision agriculture, thereby saving both time and cost [3,13,22].

Several reviews have explored the use of optical and machine learning approaches for soil nutrient analysis. Jain et al. [24] presented a critical systematic review on soil nutrient prediction using spectral data, primarily focusing on integrating multispectral and hyperspectral imaging with various machine learning algorithms. Ameer et al. [3] provided a broad overview of different analytical techniques including optical, electrochemical, and remote sensing methods for soil NPK, outlining their comparative advantages and limitations, but did not address spectral model development or data integration. Barra et al. [25] focused on preprocessing methods and chemometric methods used in soil spectroscopy, emphasizing mid- and near-infrared spectral modeling for general soil diagnostics, albeit with limited reference to real-time field application.

**Figure 1:**
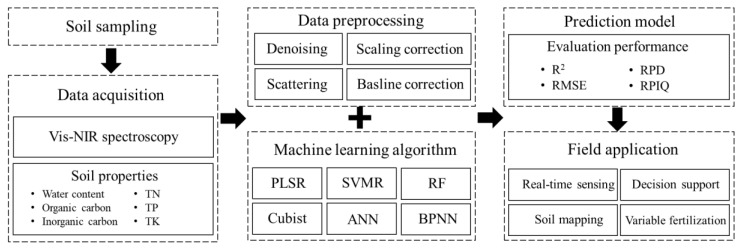
Schematic overview for evaluating soil properties using Vis-NIR spectroscopy with spectral preprocessing and machine learning (Vis-NIR, visible–near-infrared; TN, total nitrogen; TP, total phosphorus; TK, total potassium; PLSR, partial least squares regression; SVMR, support vector machine regression; RF, random forest; ANN, artificial neural network; BPNN, backpropagation neural network; R^2^, coefficient of determination; RMSE, root mean square error; RPD, residual prediction deviation; RPIQ, ratio of performance to interquartile distance).

In contrast to previous reviews that addressed integrating multispectral and hyperspectral imaging, this review specifically focused on applying Vis-NIR spectroscopy, emphasized integrating spectral preprocessing and machine learning, and explored real-time field applications under-represented in the earlier literature. An overview of evaluation framework for soil properties using Vis-NIR spectroscopy, including the steps from spectral data acquisition to field application, is schematically presented in Figure 1. Therefore, this review aimed to provide a comprehensive overview of methods for evaluating soil properties using Vis-NIR spectroscopy combined with machine learning. In particular, the review focused on (1) spectral data preprocessing methods for enhancing signal quality, (2) machine learning approaches for developing accurate prediction models, and (3) case studies related to the field application of Vis-NIR-based soil nutrient sensing. Using spectral data to estimate soil NPK content will contribute to sustainable agriculture by enabling efficient and precise nutrient management.

## 2. Research Methodology

**Figure 2:**
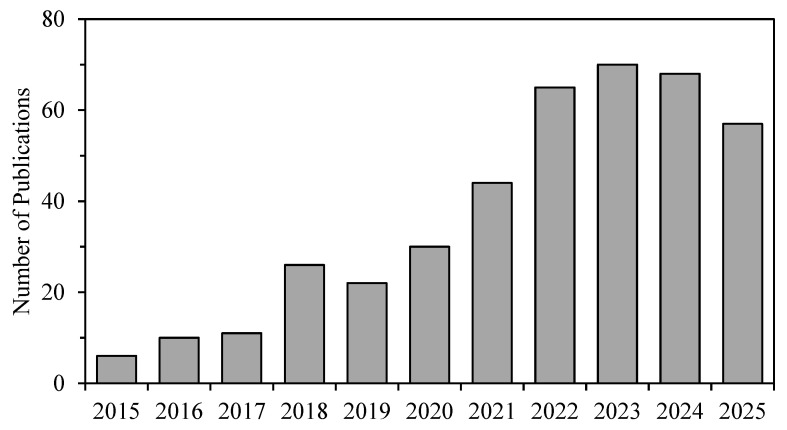
The number of publications applying machine learning to Vis-NIR spectral data for estimating soil properties.

A comprehensive literature search was conducted using the Web of Science (WoS), Scopus, and Google Scholar to identify studies applying Vis-NIR spectroscopy and machine learning for estimating soil properties. The following search string was used: (“soil” OR “soil property” OR “soil nutrient”) AND (“NIR” OR “near infrared” OR “Vis-NIR” OR “visible and near infrared”) AND (“machine learning” OR “deep learning” OR “artificial intelligence”). Articles published between 2015 and 2025 were considered. After removing duplicates, 681 articles were retained for screening. Studies were selected based on predefined inclusion and exclusion criteria summarized in Table 1. Based on the inclusion and exclusion criteria, a total of 409 studies were identified related to the research topic. The research trend on machine learning-based application of Vis-NIR for estimating soil properties from 2015 to 2025 is illustrated in Figure 2 with a steady increase over time, reaching a peak of 70 publications in 2023. Most of the studies focused on the quantification and classification of soil properties, including SOC, TN, nutrients, and texture. Several studies also showed improved analytical approaches such as the combination of Vis-NIR with imaging or X-ray fluorescence techniques and the application of various transfer method for spectral data processing.

## 3. Visible–Near-Infrared Reflectance Spectroscopy

Near-infrared spectroscopy includes the spectral region of 12,500–4000 cm^−1^ (800–2500 nm), where both electronic transitions and the overtone or combination bands of normal vibrational modes are observed [26]. Although NIR spectroscopy involves both electronic and vibrational transitions, it is difficult to distinguish the NIR region from the visible region in terms of electronic spectroscopy due to the seamless continuity between the two regions in electronic spectra [27]. Vibrational spectroscopy is based on the interaction between molecules and the electric field components of incident light in the mid- and near-infrared regions, causing the molecule to absorb light when the incident light energy (*E_P_*) is equal to the energy difference between the quantized energy levels of the vibrational states of the molecule [10]. This relationship is described in Equation (1), where ν is the frequency of incident light, c is the velocity of light, λ is the wavelength, and h is Planck’s constant:

(1) $$E_{P}=h\nu =\frac{hc}{\lambda }$$

The energy difference is determined by the chemical bonding of the functional groups in molecules that must undergo a change in dipole moment to absorb IR light [10]. The molecule can only have discrete energy levels ($E_{\upsilon }$) defined using Equation (2), where υ is the vibrational quantum number:

(2) $$E_{\upsilon }=\left(\upsilon +\frac{1}{2}\right)h\nu$$

While most molecules exist in their fundamental vibrational energy state (υ=0) at ambient temperature, atoms or atomic groups involved in a chemical bonds oscillate relative to one another at frequencies determined by the bond strength and atomic mass [28]. When the chemical bond is relatively weak or the atoms are heavy, these vibrations may occur at very low frequencies. As a result, the higher overtones in the NIR region may not be detected [10]. The functional groups containing hydrogen atoms, such as O-H, C-H, and N-H, play an important role in the chemical reactions, and the vibration of these functional groups dominates the NIR spectra, which can greatly reflect even subtle changes in molecular structure and interactions [26]. Therefore, NIR spectroscopy is advantageous for studying molecular structure and interactions. The electronic transitions observed in the near-infrared region, such as d–d transitions, charge-transfer, and π-π transitions, are typically weak and forbidden. The weak overtone and combination bands result in low absorption intensity. This characteristic distinguishes NIR spectroscopy from other domains and requires using chemometric methods to extract meaningful information [12,26].

Vis-NIR spectroscopy has been widely applied in various physicochemical and biological research fields, including food processing, agriculture, and medical diagnostics, as well as skeletal muscle studies [29,30,31,32]. In particular, Vis-NIR spectroscopy plays a crucial role in sustainable agriculture. To prevent excessive fertilization and achieve sustainable agriculture through proper nutrient management, the real-time monitoring of soil nutrients is essential [33]. Vis-NIR spectroscopy has shown great potential for assessing soil properties such as N and organic matter content, which are major indicators of soil fertility, thereby measuring and monitoring the spatial variability in soil nutrients [34].

In addition to Vis-NIR spectroscopy, there are various methods for measuring soil nutrient content, including electrochemical sensors, ion-selective electrodes (ISEs), electrical conductivity (EC) sensors, and remote sensing [3,6,35]. Electrochemical sensors offer cost-effectiveness, long-term deployability, a fast response, and multiplexing for soil nutrient measurement. However, signal drifting over time caused by imbalanced electrode surface reactions and electrode contaminations can be a significant issue [36]. ISEs, a type of electrochemical sensor, are suitable for developing portable devices because of their high selectivity and low power consumption. However, limitations such as batch-to-batch response variability, sensitivity to pH fluctuations, and the need for frequent calibration remain [33]. Although EC sensors are positively related to nitrate and exchangeable K contents and support soil nutrient prediction, EC sensors are more suitable for identifying nutrient depletion or the need for additional treatment, rather than serving as an absolute standard for nutrient evaluation due to their limited ability to distinguish specific nutrients [5,6]. Remote sensing is widely applied using ground platforms, airplanes, and satellites and is effective for large-scale monitoring. However, estimating soil nutrients with high spatial precision is challenging due to limitations in spatial resolution for capturing small-scale variability [37].

The spectrophotometer designed to be highly portable as an optical sensor provides a full spectrum in less than 1 s [38]. Compared to electrical sensing methods, Vis-NIR spectroscopy not only offers advantages such as high sensitivity, selectivity, repeatability, and immunity to electromagnetic interference but also provides the high flexibility required to simultaneously estimate multiple soil properties across diverse environmental and soil conditions [39]. By integrating appropriate spectral preprocessing and machine learning to account for spectral variability induced by environmental factors such as soil water content and temperature, Vis-NIR spectroscopy can be effectively applied for on-site soil nutrient assessment [33].

The NIR region is not as straightforward as the fundamental region because many overtone and combination bands overlap [26]. Artifacts, which represent the distortion of data, may originate from the characteristics of the measuring device, the measurement or corrupting process, and the sample itself, so they are removed through preprocessing [40]. Detailed analysis of spectra requires using techniques such as derivative spectra and chemometric methods. Chemometrics based on mathematics and statistics include various procedures for experimental design, multivariate classification, and calibration to extract detailed and meaningful chemical information from spectral data, improving the accuracy of the analytical results [26,41]. A more detailed explanation of spectral preprocessing is provided in Section 3. After artifacts are removed and the spectral data are improved through appropriate preprocessing methods, data modeling is performed to train predictive models. The application of machine learning algorithms to spectral data analysis contributes to saving both time and cost [42]. Machine learning has been widely used to handle high-dimensional and multivariate data with complex and nonlinear relationships [19]. Therefore, these algorithms can learn patterns of spectral data and perform predictive models for multiple soil properties. The predictive performance of machine learning models is evaluated using the coefficient of determination (R^2^), root mean square error (RMSE), residual performance deviation (RPD), and ratio of performance to interquartile distance (RPIQ). In general, low RMSE and high R^2^, RPD, and RPIQ values represent high predictive accuracy [15,43]. The machine learning algorithms used for modeling soil property prediction using Vis-NIR spectroscopy are provided in Section 5.

## 4. Preprocessing Spectral Data

Various factors such as instrument configuration, environmental conditions, and sample handling cause measurement errors [44]. In particular, in soil analysis, soil properties such as moisture content and texture significantly influence the NIR spectra. Therefore, appropriately applying preprocessing techniques is essential to ensure reliable and accurate spectral analysis. Engel et al. [45] reported that selecting the preprocessing strategy from the four typical categories—denoising, baseline correction, scattering correction, and scaling—can lead to performance differences of up to 20%. Bian et al. [46] tested 120 different preprocessing combinations on oil of corn, cholesterol of blood, and edible blend oil datasets by selecting one method from each of the following categories: baseline correction, scatter correction, smoothing, and scaling. Applying optimal methods led to an increase in the correlation coefficient (R) and decrease in RMSE from 0.761 to 0.902 and from 0.118 to 0.076 for the corn dataset, from 0.731 to 0.835 and from 31.33 to 25.25 for the blood dataset, and from 0.898 to 0.958 and from 0.062 to 0.047 for the edible blend oil dataset, respectively, compared to the model without preprocessing. Both studies demonstrated that combining preprocessing techniques effectively enhanced model performance, highlighting the importance of establishing a systematic preprocessing strategy. On the other hand, some combinations showed lower performance than the model without preprocessing. Vestergaard et al. [47] developed models to predict soil properties and showed that models using a single preprocessing method sometimes performed better than those with a combination of preprocessing methods such as baseline correction, scatter correction, smoothing, and scaling [47]. The research emphasized the importance of establishing an optimal preprocessing strategy, indicating that using an inappropriate preprocessing strategy may affect negatively model performance. Therefore, it is important to select and combine preprocessing methods appropriately by considering the shape of spectra data and comparing the performance of each combination of preprocessing methods.

### 4.1. Denoising

Noise refers to random fluctuations in signal amplitude across different spectral points originating from instrumental factors or sample characteristics [48]. From a signal processing perspective, such noise is typically regarded as high-frequency components; therefore, most NIR preprocessing methods apply low-pass filters, commonly known as smoothing, to perform denoising [49]. Commonly used methods for denoising include moving averaging (MA) and the Savitzky–Golay filter (SG). MA is a time series smoothing technique that analyzes data by averaging multiple subsets of the entire series. This method is employed to identify long-term trends in time series data by mitigating the effect of short-term noise [50]. Al-Mbaideen [51] reported that the most important parameter of MA is the filter length, which determines the level of smoothing. While MA acts as a low-pass filter, it has limited performance in the frequency domain, leading to the poor separation of frequency components. Ditcharoen et al. [52] developed a linear discriminant analysis (LDA)-based model for classifying durian maturity using the ratio of dry weight to initial weight. The model achieved 100% classification accuracy by applying MA combined with the standard normal variate (SNV) and baseline offset.

SG smooths NIR signals by removing high-frequency noise through polynomial regression on windowed data. The amount of smoothing depends on the window size and the polynomial order. This process is implemented as a convolution operation using polynomial regression weights as the kernel. It is commonly applied to NIR data where path length variations occur due to different particle sizes in soil samples [45,53]. Shi et al. [54] developed a PLSR-based model to predict chromium (Cr) content in soil and compared the model performance with and without applying the SG. The model with the SG showed better predictive performance (R = 0.722, RMSE = 11.66 mg kg^−1^) than the one without it. Xu et al. [55] constructed SG-based PLSR, a wavelet neural network (WNN), and SVMR models for predicting soil organic carbon (SOC) content. While the combination of SG and logarithmic transformation (Log T) was effective for the PLSR (R^2^ = 0.88, RMSE = 0.43%, RPD = 2.33) and WNN (R^2^ = 0.83, RMSE = 0.48%, RPD = 2.09) models, SG with 1D showed the strongest prediction accuracy in the SVMR (R^2^ = 0.92, RMSE = 0.36%, RPD = 2.81) model. In addition, Heil and Schmidhalter [56] compared combinations of preprocessing methods, including SG, SNV, and MSC, with the SVMR model for predicting C and N contents in soil. Applying an SG-based derivative method showed the lowest prediction error. Although the SG is an effective denoising method for spectral data, its performance is highly dependent on the selected window size and polynomial order. Inadequate parameter tuning may lead to the over-smoothing or distortion of informative spectral features. Therefore, using SG for denoising should be validated on a case-by-case basis before application.

While MA and SG are widely recognized as denoising methods, inappropriate or excessive application may inadvertently eliminate key spectral features, thus compromising model performance. Advanced signal decomposition techniques such as wavelet transform (WT) and empirical mode decomposition (EMD) have emerged as promising alternatives for effective noise reduction. Unlike the traditional Fourier transform (FT), the WT allows simultaneous signal decomposition in both the frequency and time domains, enabling more effective noise separation in spectra with abrupt signal changes. The EMD is an adaptive spectral decomposition method that does not require predefined basis functions, unlike WT approaches [57,58]. However, due to their computational complexity and lack of standardization, these methods remain challenging. Ultimately, the choice of denoising method should be guided by a data-driven, customized approach that considers sample characteristics, noise sources, and the specific analytical objectives.

### 4.2. Scattering Correction

Various parameters such as sample particle size, particle distribution, packing density, shape, and path length strongly influence the spectrum. For example, the particle size distribution leads to different intensity values even for chemically identical samples [27]. Scattering correction is usually performed by comparing a spectrum to a reference and dividing it by an estimated scatter constant [45]. Scattering correction methods include SNV, multiplicative scatter correction (MSC), and Log T.

SNV normalizes each spectrum by subtracting its mean and dividing by its standard deviation to reduce scatter effects caused by particle size and variation in light path [59]. Each spectrum is centered and scaled by the corresponding standard deviation [60]. As a result, the spectrum has an average of 0 and a standard deviation of 1 [61]. This method is often used on spectra where baseline and pathlength changes cause differences between otherwise identical spectra. It is commonly used because it adjusts each spectrum individually [62]. Hayati et al. [63] developed models for predicting the fat and moisture contents of cocoa bean using reflectance spectra. Applying SNV to the PLSR model led to improved performance (R^2^ = 0.79, RMSE = 0.79%, RPD = 2.79 for fat content; R^2^ = 0.85, RMSE = 0.43%, RPD = 2.97 for moisture content) compared with PLSR model using raw spectra (R^2^ = 0.67, RMSE = 1.17%, RPD = 1.81 for fat content; R^2^ = 0.72, RMSE = 0.64%, RPD = 1.97 for moisture content). In addition, for the freshness assessment of preserved eggs, applying SNV to both PLSR and SVMR algorithms increased R^2^ from 0.82 to 0.87 and from 0.77 to 0.91 and decreased RMSE from 1.74 to 1.49 mg kg^−1^ and from 1.97 to 1.27 mg kg^−1^, respectively.

MSC adjusts the scattering of each spectrum by fitting it to the average spectrum of all samples through the least squares method [64]. It assumes that noise arises from both multiplicative and additive biases, which are corrected by linearly regressing each spectrum against a reference, thereby effectively reducing spectral distortions [53]. While MSC has the drawback of requiring entire sample data, pretreatment is effective at minimizing baseline offsets and multiplicative effects [62]. This method supports the prediction performance of fat and moisture contents in cocoa bean using the PLSR algorithm. The MSC-based PLSR model showed an increase in R^2^ and a decrease in RMSE by 20.90% and 34.45% for the fat content and 18.06% and 32.81% for the moisture content, respectively [63]. Masithoh et al. [65] built a PLSR-based model to predict the content of coconut sugar, an impurity found in *Arenga pinnata* sugar. Applying MSC significantly improved model performance, increasing the R^2^ by 0.31 and reducing RMSE by 8.54% compared to the model based on raw spectra. As noted by Rinnan et al. [66], SNV and MSC perform similarly; thus, it is important to apply them based on the characteristics of the data. SNV treats each spectrum independently, making it potentially advantageous in cases with significant baseline variations. In contrast, MSC relies on the mean of the entire dataset, which could reduce its robustness when there are large differences among samples.

Log T not only converts transmittance or reflectance data into absorbance for physical interpretability but also enhances the visibility of spectral features and reduces the influence of multiplicative variations caused by lighting or instrumental conditions [66,67]. Transforming the reflectance spectrum into the absorbance spectrum using Log T sometimes leads to improved performance. Rahmawati et al. [68] developed a model to predict white rice flour adulteration in brown rice flour using the PLSR algorithm and compared the performances of models based on the reflectance spectrum, Kubelka–Munk (KM) modified spectrum, and Log T modified spectrum. With the application of SG-based 1D, the Log T-modified spectrum showed the best performance (R^2^ = 0.88, RMSE = 6.05% in reflectance spectrum; R^2^ = 0.82, RMSE = 7.45% in KM-modified spectrum; R^2^ = 0.93, RMSE = 4.56% in Log T-modified spectrum).

Conventional scattering correction methods still need to evolve through sustained research and refinement due to their several limitations. Current methods are generally considered reliable only when the chemical differences between the sample spectrum and the reference spectrum are negligible. Recognizing these inherent drawbacks, Li et al. [69] proposed three improved approaches that integrate the first-order derivative (1D), linear regression correction (LRC), and orthogonal signal projection (OSP) based on two conflicting assumptions about the relationship between addition coefficients and wavelengths. In the study, the proposed methods achieved better performance, showing a lower RMSE than traditional approaches such as SNV and MSC in the apple, meat, and mixture of gluten and starch powder datasets. Wan et al. [70] highlighted that many existing scattering correction techniques rely on assumptions and experiences during preprocessing, leading to overfitting during spectral modeling. They also noted the fundamental limitation arising from the absence of a mathematical function that accurately describes the nonlinear relationship between the spectral signal and the analyte.

### 4.3. Baseline Correction

Baseline errors, including drift and distortion, are critical factors that undermine the reliability of spectral data. They often arise from instrumental instabilities such as fluctuations in light source temperature, changes in mirror angle, and shifts in laser wavelength [71]. They manifest in various forms, including offset and sloped or curved baselines in the spectrum [45,71].

Derivatives are primarily used to resolve peak overlaps and to eliminate constant and linear baseline drift between samples. Spectral derivatives can be calculated by taking the differences between two consecutive points or by smoothing and differentiating over a specified gap distance [62]. A 1D eliminates constant baseline offset and a second-order derivative (2D) can also eliminate slope [60]. Delgadillo-Duran et al. [72] developed regression models to predict soil properties using NIR spectra. Spectral data preprocessed with 1D and 2D showed R^2^ values of 0.55 for Ca, 0.44 for Mg, 0.38 for OM, and 0.81 for pH prediction, respectively. Guo et al. [73] reported that the 1D-based model performed better for reducing the interference of moisture in predicting N content in fresh tea leaves, as indicated by an increase in R^2^ from 0.58 to 0.69 and a decrease in RMSE from 1.51% to 0.12%. Despite the 1D and 2D being effective methods for spectral data, integer-order derivatives such as 1D and 2D lack sensitivity to gradual tilts or curvatures that contain meaningful information. Recent studies emphasized the need for employing more versatile and adaptive methods, given that spectral data often display diverse and complex characteristics. Hong et al. [74] suggested using the fractional-order derivative (FOD) method in the SVMR model for analyzing organic matter. In this study, for the full spectrum, the 1.5-order derivative spectra showed the best performance, with R^2^ = 0.79 and RMSE = 4.67%, while raw spectra, 1D spectra, and 2D spectra showed R^2^ and RMSE values of 0.55 and 6.75%, 0.69 and 5.84%, and 0.72 and 5.53%, respectively. Lao et al. [75] also found that using 0.75-ordered derivative spectra enhanced prediction performance for salinized soil moisture content, with R^2^ increasing by 17.1%, 6.41%, and 80.4% and RMSE decreasing by 24.4%, 11.5%, and 44.1% compared to the ELM models using raw spectra, 1D spectra, and 2D spectra, respectively. This highlights the importance of selecting an appropriate derivative order.

Continuum removal (CR) is effective at isolating specific absorption features and eliminating the influence of varying slopes and overall reflectance [76]. This method is performed by dividing or subtracting the raw spectrum by its continuum curve, which is obtained using the convex hull method or polynomial fitting [21,76,77]. Guo et al. [73] reported that the R^2^ of 0.58 for raw spectrum model increased to 0.67 for the CR, and the RMSE decreased from 1.51 to 0.11%, which is similar to that for the 1D-based model. Continuum removal also improved the prediction performance of lead (Pb) and cadmium (Cd) contents in metal mixture-spiked soils. Compared with raw spectra, applying CR resulted in an increase in R^2^ from 0.80 to 0.88 and a decrease in RMSE from 240.9 to 99.9 mg kg^−1^ for Pb content prediction. For Cd content prediction, R^2^ increased from 0.52 to 0.74 and RMSE decreased from 4.99 to 2.29 mg kg^−1^ [78]. When applying CR, careful baseline construction is required because an inaccurate continuum line may distort the true spectral features. Since the baselines might vary depending on the instrument status and the analytical conditions related to sample properties, CR optimization is required for each spectral dataset [79].

### 4.4. Scaling

Scaling is a preprocessing technique used to equalize the contribution of each peak by minimizing unit differences and interpretive bias, often through normalization or adjusting the spectral area. Autoscaling (AS), commonly used for scaling, involves centering each variable to its average and then dividing by its standard deviation. This method helps to balance variable influence; however, it inadvertently amplifies the importance of variables that contain mostly noise due to their low variance [45]. Huang et al. [80] reported the effects of four preprocessing methods, namely original, Log T, AS, and Log T combined with AS, on the prediction of various tomato firmness parameters using acoustic, impact, compression, and puncture tests. Overall, with a few exceptions, AS improved model performance by increasing the R^2^ by 0–16.2% and decreasing the RMSE by 0.04–18.9% compared to other preprocessing methods. In addition, other scaling techniques such as mean centering (MC), max–min scaling (MMS), and Pareto scaling (PS) are also commonly applied depending on the characteristics of the data [46,81,82]. MC shifts the mean to zero by subtracting the average, while MMS transforms the data to fit a range between 0 and 1, preserving the original relationship. PS centers the variables by subtracting the mean and then divides each variable by the square root of its standard deviation, enhancing the contributions of variables with smaller standard deviations. Scaling methods are sometimes considered unnecessary because other preprocessing techniques indirectly produce similar effects. For instance, SNV inherently provides a normalization effect. However, since the primary purpose of SNV is to remove physical phenomena affecting the spectra, it could be different from statistical normalization [66]. Therefore, in certain cases, applying scaling methods to balance the relative influence of variables contributes to improved model performance.

## 5. Determination of Wavelength Range

Dimension reduction methods with wavelength selection are powerful tools for not only extracting valid information from raw spectral curves but also improving the potential of spectroscopy to effectively solve the multicollinearity problem between spectral wavelengths [83]. Wavelength can be selected based on the theory of the molecular bond energy of each chemical compound or data value, such as the contribution of each value to model performance.

### 5.1. Theory-Based Determination

Each molecular bond exhibits characteristic energy levels, resulting in absorption peaks at specific wavelengths, as shown in Table 2. These distinct spectral features enable identifying and analyzing chemical substances based on their molecular structure [84]. Hydrocarbon bonds are observed between 600 and 3300 cm^−1^ due to the C-H stretching vibration. In particular, C-H sp^3^, C-H sp^2^, and C-H sp are shown around 3333–3571 nm, 3226–3333 nm, and 3030 nm, respectively. The C≡N and C≡C triple-bonds of nitriles and alkynes appear at 4348–4545 nm, and O-H or N-H bonds of alcohols and amines appear at 2703–3333 nm. The C=O of carbonyl stretch can be found at 5495–6098 nm, and the C-O stretch of ethers appears at 7937–9523 nm [85]. Many peaks related to soil components are observed in Vis-NIR and MIR regions. Soil organic matter shows various peaks for alkyls, protein amide, carboxylic acid, carboxylate anion, and aromatic groups appearing at 3413–3509 nm, 5952 and 6535 nm, 5814 nm, 6250 and 7143 nm, and 6250–6369 nm, respectively. The influence of water appears at 6135 nm [11].

### 5.2. Data-Based Determination

Wavelength selection can be performed through statistical methods such as principal component analysis (PCA), the successive projections algorithm (SPA), the simulated annealing algorithm (SA), and competitive adaptive reweighted sampling (CARS). Table 3 presents the detection wavelengths of each substance according to the applied selection method.

Principal component analysis (PCA) groups variables with correlated information into principal components (PCs). Initial PCs account for the key variances, mainly related to chemical features, while the subsequent PCs account for the lower variances, mainly indicating noise [86].

The successive projections algorithm is a method that arranges variables, evaluates subsets of these variables, and reduces dimensionality by removing uninformative variables [87]. The simulated annealing algorithm is a stochastic optimization algorithm inspired by metal annealing that avoids becoming trapped in local minimum by allowing probabilistic jumps that decrease over time [88]. The competitive adaptive reweighted sampling algorithm uses absolute PLSR coefficients to assess the importance of each variable. Based on the importance of variables, it iteratively selects wavelength subsets via Monte Carlo sampling and selects the most relevant variables using the double-step wavelength selection procedure based on the regression coefficient [89].

Beattie et al. [86] showed that PCA revealed correlations between fatty acid composition and wavenumbers. By compressing complex spectral variations into a small number of direct variables, they showed that accurate predictions were possible even with limited data. Guo et al. [17] developed prediction models for available potassium (AK), available phosphorus (AP), and soil organic matter (SOM) of soil using CARS and SPA. The peaks selected for AK were associated with various compounds such as ferrihydrite, goethite, and amine. AP showed similar associations to those of AK, involving diverse and complex features. SOM was related to the overtones and combination absorption of O-H, C-H, and N-H bonds. Wang et al. [88] demonstrated that dimension reduction via wavelength selection significantly improved the prediction performance for AK compared to using raw spectral data. Each selection method showed different peak ranges. SPA selected 22 peaks within the ranges of 400–543, 709–800, 1230–1384, 1558–1730, and 3330–3990 nm. SA selected 40 peaks, primarily located within the ranges of 1359–1442, 2158–2420, and 2864–3498 nm. CARS selected 49 peaks within the ranges of 715–873, 1024–1263, 1406–1629, 3012–3334, and 3595–3732 nm. Each RAW–PLSR, SPA–PLSR, SA–PLSR, and CARS–PLSR model showed R^2^ values of 0.39, 0.49, 0.49, and 0.62 and RMSE (mg kg^−1^) values of 55.32, 22.8, 34.2, and 32.1, respectively. Cheng et al. [18] also reported variable selection techniques for estimating soil total nitrogen using the PLSR, SVR, BPNN, and ELM models. In this study, CARS, GA, and SPA were employed as variable selection techniques, and 32 peaks were chosen within the ranges of 400–850 and 1900–2400 nm by CARS. GA selected 37 peaks and SPA selected 66 peaks within ranges around 1380, 1450, 1580, and 1700 nm. Although SPA exhibited lower performance than using raw spectra, CARS and GA performed better than the models based on raw spectra (when averaged across all models, R^2^ = 0.61 and RMSE = 0.22 g kg^−1^ in raw spectra, R^2^ = 0.74 and RMSE = 0.18 g kg^−1^ in CARS, R^2^ = 0.67 and RMSE = 0.2 in GA, and R^2^ = 0.49 and RMSE = 0.24 g kg^−1^ in SPA).

**Table 3:** The selected wavelength ranges for nutrients based on the statistical methods.

Nutrients	Wavelength Selection	Selected Wavelength (nm)	Reference
Available K	SPA	400–421, 996, 1350, 1351, 1680, 2372, 2448	[17]
Available P		400–436, around 1000, 1325–1417, 1604, 1659, 1835~1946, 2355–2450	
Soil organic matter		405–442, 543~788, around 1000, 1295, 1835–1934, 2210	
Available K	CARS	405–483, around 728, 967~1031, 1271–1409, 1643–1789, 1975–2004, 2109–2174, 2312–2449	
Available P		400–450, 1005–1083, 1292–1358, around 1577, 1964–2044, 2113–2216, 2381–2421	
Soil organic matter		411~508, 984–1028, around 1233, 1347–1358, 1608~1620, around 1836, 1930–2052, 2309–2448	
Organic carbon	CARS-PLSR	450, 520–535, 560–575, 630–640, 1895–1905, 2210, 2495–2500	[90]
Nitrogen		515, 570–575, 660–665, 1880–1890, 2205–2210	
Available K	SPA	400–543, 709–800, 1230–1384, 1558–1730, 3330–3990	[88]
	SA	449–876, 1359–1442, 2158–2420, 2864–3498, 3618–3982	
	CARS	715–873, 1024–1263, 1406–1629, 3012–3334, 3595–3732	

CARS, competitive adaptive reweighted sampling; PLSR, partial least-squares regression; SA, simulated annealing algorithm; SPA, successive projections algorithm.

## 6. Machine Learning Algorithms for Predicting Soil Properties

Artificial intelligence (AI) is essential for effectively predicting soil properties through Vis-NIR spectral data. Machine learning, as a subset of AI, develops algorithms and statistical models to learn from data and improve task performance [24]. Integrating chemometrics and machine learning enables the investigation of relationships between chemical parameters and the measured instrumental signals [25]. Machine learning analyzes and interprets complex spectral data patterns. Machine learning performs better in data regression with nonlinear relationships, which are commonly found in agricultural systems [91]. Given the diversity of spectral data and soil properties, the choice of machine learning algorithm is critical in predicting model performance. Since each machine learning algorithm has different capabilities in handling the complexity and noise of spectral data and overfitting issues, selecting an appropriate model is essential for optimizing predictive accuracy.

Various machine learning algorithms have been employed to predict soil properties using Vis-NIR spectral data, each with distinct characteristics. Table 4 summarizes the characteristics and limitations of different machine learning algorithms.

Machine learning model performance is evaluated using performance metrics, including the coefficient of determination (R^2^), root mean square error (RMSE), residual performance deviation (RPD), and ratio of performance to interquartile distance (RPIQ). The performance metrics are defined using Equations (3)–(6), where $Y_{i}$ is the predicted value, $y_{i}\;$ is the observed value, $\bar{y}\;$ is the average value of $y_{i}$, and n is the number of samples. The R^2^ indicates how much of the variation in the dependent variable is explained by the linear regression model. Its value ranges from 0 to 1, where 1 signifies perfect explanation of the variance and 0 indicates none of the variances [102]:

(3) $$R^{2}=1-\frac{\sum_{i=1}^{n}{{(y}_{i}-Y_{i})}^{2}}{\sum_{i=1}^{n}{{(y}_{i}-\bar{y})}^{2}}$$

The RMSE represents the average deviation between the observed and predicted values, with smaller RMSE values indicating higher model accuracy and zero representing a perfect fit [24].

(4) $$RMSE=\sqrt{\frac{1}{n}\sum_{i=1}^{n}{\left(Y_{i}-y_{i}\right)}^{2}}$$

The RPD is used to evaluate the model accuracy by analyzing the relationship between standard deviation (SD) and RMSE. If the RPD value is below 1.5, the model performance is considered to be very poor, suggesting that the predictions were unsuccessful. If the RPD value is between 1.5 and 1.8, the model is considered to provide acceptable results, and an RPD value from 1.8 to 2 indicates good model performance. An RPD value greater than 2 indicates excellent predictive performance [19].

(5) $$RPD=\frac{SD}{RMSE}=\sqrt{\frac{\frac{1}{n-1}\sum_{i=1}^{n}{\left(y_{i-}\bar{y}\right)}^{2}}{\frac{1}{n}\sum_{i=1}^{n}{\left(Y_{i-}y_{i}\right)}^{2}}}=\frac{1}{\sqrt{\left(1-R^{2}\right)}}$$

The RPIQ evaluates model performance by calculating the ratio between the interquartile range of the observed value and the RMSE. $Q_{1}$ and $Q_{3}$ represent the values below which 25% and 75% of the data fall, respectively. A higher RPIQ value indicates excellent predictive performance [43].

(6) $$RPIQ=\frac{Q_{3}-Q_{1}}{RMSE}$$

Among the commonly used machine learning methods for predicting soil properties, the PLSR and SVM demonstrated strong performance in predicting SOC in the top 10 cm of soil with R^2^ values above 0.80 and RPD values exceeding 2.5 [103]. This enhanced performance of the SVM, particularly the higher SOC content, has been attributed to its capacity to model complex and nonlinear relationships in high-dimensional spectral data [103]. Bai et al. [104] reported that among SOC prediction models such as the one-dimensional CNN (1-CNN), two-dimensional CNN (2-CNN), long short-term memory network (LSTM), and deep belief network (DBN), the 1-CNN combined with the interval random frog showed the best performance (R^2^ = 0.90, RMSE = 0.15, RPIQ = 4.20).

Machine learning-based models have also been effectively applied to predict soil nutrients. For soil available N, P, and K, the PLSR model performed better than the SVMR with R^2^ values over 0.7 [7]. The 1-CNN achieved a higher R^2^ value of 0.88 and an RPIQ value of 2.49 for predicting oxalate extractable-P compared to the PLSR and RF [105]. In addition to its high predictive accuracy, the 1-CNN model demonstrated the lowest prediction bias and consistent performance across different land-use systems, indicating its robustness and generalizability. Although the ELM model performed better than the PLSR, SVMR, and BPNN at predicting TN content, the accuracy was moderate, with an R^2^ value of 0.65 [18]. The limited performance may be attributed to structural limitations such as the risk of overfitting due to excessive hidden nodes and instability caused by non-optimal or unnecessary weight values and thresholds [18]. Cubist, another model frequently used for predicting soil properties, demonstrated good performance in estimating soil water content with R^2^ values above 0.70 [106]. These results highlight the potential of machine learning-based models for assessing soil properties using Vis-NIR spectroscopy. Continued evaluation and comparison using appropriate performance metrics are expected to further improve and optimize these prediction models.

However, practical deployment in the field requires a broader perspective. Factors including interpretability, computational cost, and adaptability to diverse soil types should also be considered. While the SVMR and CNN may provide higher predictive accuracy, PLSR or Cubist could be more feasible for rapid or on-site assessments due to their lower computational demands and easier interpretability. The prediction performance of machine learning models often varies depending on the target soil property. Since C compounds have stronger and more distinct absorption features in the Vis-NIR region, SOC is generally predicted with higher accuracy than N. Although prediction models may yield high R^2^ or RPD values, these performance metrics do not always reflect reliable performance under diverse field conditions or soil management strategies. To overcome these limitations, recent studies have studied ensemble-based models. In a previous study comparing of deep learning architectures applied to Vis-NIR spectra, the ensemble approach yielded comparable or superior predictive accuracy [107].

Ke et al. [108] reported a six-layer CNN architecture with an encoder–decoder structure for improving the prediction performance for seven soil properties, achieving R^2^ values over 0.93. Liu et al. [109] combined fractional-order derivatives with a 1-CNN, effectively addressing soil moisture interference and improving prediction accuracy for soil organic matter. Wang et al. [110] introduced a multi-gate mixture-of-experts network, incorporating feature-specific pathways and data augmentation, thus outperforming conventional CNN and long short-term memory (LSTM) architectures. These approaches demonstrate that structurally integrated models improve generalization across heterogeneous soils and minimize dependence on the strengths or assumptions of individual models. Multi-attribute prediction accuracy has been improved using dual-stream convolutional models that integrate one- and two-dimensional spectral features [111], as well as through localized multi-channel CNNs that leverage neighborhood-based error correction [112]. Wang et al. [113] applied a memory-based learning approach using neighbor (*k*) adaptation, showing superior performance over conventional models in predicting multiple soil properties, particularly when *k* was optimally tuned. These approaches represent significant advances in addressing spectral heterogeneity and enabling accurate simultaneous prediction of multiple soil properties.

## 7. Application of Vis-NIR Spectroscopy for Prediction of Soil Properties

### 7.1. Soil Water Content

Soil water content is a major hydrodynamic soil property and influences varied environmental processes, including plant growth, erosion, biogeochemical cycling, and water regulation [114]. Applying Vis-NIR spectroscopy for predicting soil properties is feasible because various soil characteristics influence the absorption and reflection patterns in the Vis-NIR spectrum [1]. Soil water content is also reflected in the Vis-NIR spectrum. For example, in the NIR region (700–2500 nm), absorption is mainly influenced by the presence of soil moisture, clay minerals, and soil organic matter [115]. The presence of moisture in soil samples causes specular reflectance due to overtones and the following fundamental vibrations of the water molecule: symmetric and asymmetric O-H stretching and O-H bending [15,116]. The three forms, soil water–hygroscopic, capillary, and gravitational, exhibit distinct absorption peaks near 1400, 1900, and 2200 nm [115].

Soil reflectance decreases with decreasing matric potential, attributed to the shift from capillary forces to adsorptive surface forces, corresponding to wet and dry soil conditions, respectively [117]. Since soil moisture significantly impacts reflectance, accurately predicting soil water content using Vis-NIR spectroscopy is important and contributes to improving the accuracy of predictions for other soil properties. Marakkala Manage et al. [116] evaluated Vis-NIR spectroscopy for soil water content measurement under varying matric potentials: full saturation, −10 cm (pF 1), −30 cm (pF 1.5), −300 cm (pF 2.5), −1000 cm (pF 3), and air-dry conditions. The prediction model of soil water content using PLSR achieved R^2^ values ranging from 0.74 to 0.84 and RPIQ values between 1.7 and 2.3 across all water content levels, indicating that Vis-NIR spectroscopy is reliably applied to estimate various soil water content. In a study using unmanned aerial vehicle (UAV)-based remote sensing to predict soil water content at a depth of 15 cm, visual spectrum, near-infrared and infrared/thermal imagery, and vegetation indices were used as input variables for the Bayesian artificial neural network (Bay-ANN), achieving high performance with an R^2^ of 0.85 and an RMSE of 1.1% [118]. Table 5 summarizes applications of Vis-NIR spectroscopy using machine learning for soil water content prediction.

### 7.2. Soil Organic and Inorganic Carbon

Since soil organic carbon (SOC) is important for maintaining soil health and quality, accurately estimating SOC is essential for evaluating the spatial variability in soil fertility status and ecosystem services [121]. Soil inorganic carbon (SIC) also contributes to soil functions, ecosystem services, and the global C cycle as a major component of the soil C pool in arid and semiarid regions [104]. SIC exists mainly in the form of calcium carbonate (CaCO_3_) and consists of calcite or dolomite [122]. Traditional methods for measuring SOC and SIC, such as dry combustion techniques, chemical oxidation, and acid solution extraction, are destructive, time-consuming, and costly, requiring hazardous reagents [123]. Therefore, cost-effective and non-destructive Vis-NIR spectroscopy has recently been used to predict SOC and SIC contents.

Due to the high dimensionality of spectral data, soil C prediction models are being developed by applying appropriate spectral preprocessing methods and machine learning algorithms. The PLSR models for SOC and SIC prediction in Italian calcareous soils with low SOC and high CaCO_3_ contents performed best, with SNV, SG smoothing, and 1D (SOC: R^2^ = 0.66, RMSE = 0.031%; SIC: R^2^ = 0.93, RMSE = 0.26%) [122]. Ding et al. [123] investigated SOC prediction in arid wetland soils of northwest China using Vis-NIR spectroscopy with machine learning. Among the models, i.e., RF, RF-SVM, and ant colony optimization–interval partial least squares (ACO-iPLS), the RF-SVM combined with 1D preprocessing model performed best (R^2^ = 0.91, RMSE = 0.27%, RPD = 2.41), demonstrating high accuracy for SOC estimation to support improved management in desert wetland ecosystems. Regarding the SOC content in sandy loam and loamy soils, values for the Gangetic alluvial plain were predicted using Vis-NIR spectroscopy with the PLSR and SVMR. The SVMR combined with 1D (R^2^ = 0.84, RPD = 2.47) performed better than PLSR combined with 1D (R^2^ = 0.41, RPD = 1.31) [7]. Studies that predicted soil organic and inorganic C contents using Vis-NIR spectroscopy while applying machine learning tools such as PLSR, RF, and SVM are presented in Table 6.

### 7.3. Soil Nutrients

Intensive agricultural practices used to meet growing food demands decrease soil quality, creating a need for increased productivity and environmental sustainability [91]. Since soil nutrients and properties serve as fundamental indicators of soil fertility, soil management significantly influences agricultural productivity, food security, and the sustainable agroecosystem [17,125]. Therefore, analyzing the soil nutrient content and applying fertilizer accurately are important for crop growth and sustainable agriculture. Instead of traditional soil nutrient analysis methods, which are expensive and time-consuming, rapidly and accurately assessing soil nutrients using Vis-NIR spectroscopy enables the real-time evaluation of spatial variability.

Predicting soil nutrient content using Vis-NIR spectroscopy is possible either through the direct absorption of infrared radiation by specific molecular groups such as carbonates or indirectly through correlations with infrared-active minerals such as Al, Fe, and Mn oxides [11]. Molecular vibrations induced via the interaction between light and elements such as C, N, H, O, P, and S produce reflectance spectra in the NIR region, characterized by weak overtones and combination bands in the range of 700–2500 nm [126]. Soil elements including Ca, Mg, Na, Al, Fe, P, S, Si, and Ti, when present in soil constituents such as clays, oxides, hydroxides, and sulfates, exhibit characteristic absorption bands in the Vis-NIR region [11]. Zhang et al. [103] investigated PLSR and SVM performance for predicting TN content in topsoil (0–10 cm) and subsoil (10–40 cm) using Vis-NIR spectroscopy. The SVM (R^2^ = 0.89–0.91) showed better performance than PLSR (R^2^ = 0.85–0.88), being particularly better at predicting TN in topsoil (R^2^ = 0.91) than in subsoil (R^2^ = 0.89).

For certain nutrients such as soil K and P, measurement can be challenging due to the lack of a direct spectral response and their typically low concentrations, complicating the process of indirect inversion based on other soil components [17]. Therefore, appropriate spectral preprocessing and inversion modeling are essential to identify the most suitable model for accurate prediction. Devianti et al. [15] demonstrated that combining Vis-NIR spectroscopy with various spectral preprocessing methods (e.g., autoscaling, generalized least squares weighting (GLSW), detrend + GLSW, and external parameter orthogonalization (EPO)) and machine learning algorithms (SVMR, PLS-ANN, and GBRTs) enabled the accurate prediction of TN, TP, and TK contents in cropland soils. Among the combination, the GBRTs with EPO processing yielded the highest prediction accuracy with RPD values of 2.64, 3.92, and 2.38 for TN, TP, and TK, respectively. These findings highlight the importance of selecting an optimal combination of preprocessing methods and machine learning models to enhance the accuracy of nutrient prediction across diverse soil conditions.

The available fraction of soil nutrients, referred to as plant-available nutrients, is used to assess the environmental fate and mobility of nutrients [11]. Measuring plant-available nutrients is essential for increasing crop productivity, optimizing fertilizer application rates, and monitoring soil quality over time [4,103]. Liu et al. [67] evaluated the predictive performances of various soil nutrients including TN, nitrate N, AP, and AK using the PLSR model combined with seven types of preprocessing methods: SG smoothing, 1D, 2D, SNV, MSC, normalization (NOR), and logarithmic transformation (Log T). The best prediction results were obtained using R + SG + NOR + SD for TN (R^2^ = 0.98), Log T + SG + 1D for NN (R^2^ = 0.90), Log T + SG + MSC + 2D for AP (R^2^ = 0.85), and R + SG + SNV + 2D for AK (R^2^ = 0.89). Combining appropriate spectral preprocessing methods and machine learning algorithms is an effective approach for predicting both total and plant-available nutrients in soil using Vis-NIR spectroscopy.

As summarized in Table 7, soil nutrient prediction using Vis-NIR spectroscopy has involved diverse modeling approaches. The PLSR is the most frequently used, while advanced models such as ELM, GBRTs, and SVMR have shown improved performance, particularly for TN and P. Prediction accuracy has varied across nutrients and soil conditions, with some studies achieving R^2^ values above 0.90 and RPD values over 2.5.

## 8. Field Applications of Vis-NIR Spectroscopy for Soil Nutrient Management

Although portable spectrophotometers such as ASD FieldSpec and Labspec have been used to predict soil properties such as soil water content, organic and inorganic C, and nutrients, these studies were usually conducted under laboratory conditions to ensure spectral consistency and accurate model development (Table 5, Table 6 and Table 7). To apply the developed model in the field, it is important to evaluate the on-site performances of prediction models trained in the laboratory. Zhou et al. [13] developed a detector for real-time soil TN measurement based on NIR spectroscopy with the ELM algorithm. The detector was designed with a modular concept and mounted on a tractor for on-site measurement. The laboratory calibration of the TN detector showed high accuracy (R^2^ = 0.90), and field validation also demonstrated reliable performance (R^2^ = 0.82), indicating that the detector could provide stable and accurate TN content in the field.

Since models developed from soil spectral data measured in the laboratory or from soil spectral libraries are often difficult to apply in the field, the calibration model can be transferred between sensors using transfer learning, aiming to propagate knowledge from the source domain to the target domain [127]. The RF model for predicting SOC using Vis-NIR spectroscopy was developed under laboratory conditions and applied to on-line field measurements [124]. While models based on laboratory spectra showed reduced performance when directly applied to on-line conditions (R^2^ = 0.42, RMSE = 0.27%, RPD = 1.32), applying spiking with a small number of field samples significantly improved prediction accuracy (R^2^ = 0.75, RMSE = 0.17%, RPD = 2.04). Similarly, Kok et al. [125] reported that a field spectra model transferred from a laboratory spectra model improved prediction accuracy for organic C and N, with R^2^ increasing from 0.78 to 0.80 and 0.77 to 0.82, respectively.

In addition to soil C and N, on-line Vis-NIR spectroscopy has been applied to P management. Mouazen and Kuang [23] used an on-line Vis-NIR spectroscopy system mounted on a subsoiler-based platform for measuring available P across a 21 ha cropland site in the UK for 3 years. The spectral data were preprocessed using SG smoothing and 1D, and the PLSR model was applied using the laboratory reference values of available P, achieving an R^2^ of 0.60 and an RPD of 1.50 via on-line validation. The sensor-predicted P maps were used to implement variable-rate P application. This study demonstrated the potential of on-line Vis-NIR spectroscopy for in-field P mapping and data-driven fertilization management.

## 9. Challenges and Prospects of Vis-NIR Spectroscopy Combined with Machine Learning for Soil Nutrient Prediction

Integrating Vis-NIR spectroscopy and machine learning has shown considerable promise in enhancing the efficiency and accuracy of soil property prediction. Numerous studies have demonstrated successful applications across various soil types and geographical regions. However, several critical challenges remain regarding the reliability, scalability, and on-site application of prediction models.

The use of Vis-NIR spectroscopy combined with machine learning for predicting soil property is affected by various interacting factors, such as the physical characteristics of soil samples, the spectral measurement process, and the applied machine learning algorithm. The quality and consistency of spectral data are crucial for prediction accuracy. However, soil moisture and temperature, particle size, and homogenization cause substantial noise or baseline shift. To address these issues, many studies have applied preprocessing methods such as SG, PCA, 1D, and 2D (Table 5, Table 6 and Table 7). These techniques can enhance signal quality but often lack generalizability across soil types and conditions, which may result in insufficient correction of nonlinear spectral effects. Therefore, the further development of preprocessing is needed to account for both linear and nonlinear spectral relationships, effectively remove noise, and identify the spectral wavelengths strongly correlated to target soil properties.

The predictive performance of models is strongly influenced by the strength of the spectral signature associated with the target soil property. Since available N and K present weak or indirect spectral features, the prediction accuracy tends to be lower than that of SOC (Table 6 and Table 7). To compensate for the weak spectral representation of some soil attributes, integrating auxiliary data sources may enhance model performance. Zayani et al. [128] demonstrated that combining remote sensing data with laboratory spectral data improved the prediction performance for SOC.

Machine learning algorithms are also important for improving model performance. Although linear models such as PLSR are computationally efficient and interpretable, they are often inadequate for modeling complex and nonlinear relationships. Cubist effectively accommodates both linear and nonlinear relationships through its rule-based linear regression models, but interpreting variable importance is limited [77]. CNNs have shown superior performance in extracting hierarchical spectral features in high-dimensional datasets. However, their high computational cost, demand for large labeled datasets, and limited model transparency remain significant barriers to their field-level application. Each machine learning algorithm has its own advantages and disadvantages. Ensemble approaches that integrate multiple algorithms have been increasingly applied in Vis-NIR-based soil prediction to enhance model robustness and reduce overfitting. Integrating the strengths of multiple models compensates for individual model weakness, especially when dealing with heterogeneous spectral signals or complex soil characteristics. However, for practical application, transparency, model complexity, and field applicability should be carefully considered.

The field application of Vis-NIR spectroscopy for nutrient prediction is challenging because of sensor calibration, environmental variability, and data transferability. Inconsistent sensor calibration between devices under field conditions results in variability in spectral measurements, thereby reducing the reliability of predictive models. Environmental factors such as soil moisture, temperature, and surface roughness influence soil reflectance, causing decreased model performance when laboratory-developed models are applied under field conditions. Moreover, the transferability of models across different geographic or climatic regions is a critical challenge, as models often fail to generalize when applied outside their training environments. Without appropriate domain adaptation or recalibration strategies, predictive performance may decline significantly. To address these challenges, recent studies have investigated transfer learning strategies. A hybrid transfer learning model improved SOC prediction across different regions by reducing the negative impact of regional variability [129].

Traditional methods for assessing soil properties provide high analytical accuracy but are less suited to real-time monitoring due to their time-consuming application. Vis-NIR spectroscopy combined with machine learning has the potential to simultaneously predict multiple soil properties. This makes it especially attractive for large-scale field applications and decision support in precision agriculture. Integrating Vis-NIR spectroscopy with Internet of Things (IoT) technologies and autonomous platforms such as UAV may enable in situ measurements at higher spatial and temporal resolutions and offers real-time monitoring and cloud-based data sharing. Yuan et al. [130] reported the potential of the UAV-based cluster-hybrid approach using PLSR, SVR, and a deep neural network (DNN) to generate high-resolution soil nutrient maps by adapting the prediction model to each spectrally distinct cluster. Combining Vis-NIR spectroscopy with remote sensing data from satellites could facilitate multiscale soil monitoring by linking ground-level measurements with broad-area observations. To ensure the reliable application of the techniques in the field, sensor standardization, calibration transfer protocols, and robust data fusion frameworks are required. Yang et al. [131] reported that the self-supervised transfer learning method (pyramid external attention model and masked autoencoder-based transfer learning (PEAMATL)) effectively mitigated domain shift by extracting domain-invariant features without requiring extensive labeled data.

## 10. Conclusions

For sustainable agriculture, accurately assessing soil nutrient levels and appropriate fertilizer application are essential. Since there is no direct and real-time method for determining soil nutrient levels, Vis-NIR spectroscopy has emerged as an effective approach for soil nutrient estimation. Vis-NIR spectroscopy offers the high sensitivity, selectivity, and flexibility required to simultaneously measure multiple soil properties in a non-destructive way. The preprocessed Vis-NIR spectra data can be integrated with machine learning algorithms to evaluate soil nutrient levels more accurately and rapidly. Since each spectrum is obtained under different conditions, the selection of data-specific preprocessing strategy is important. Inappropriate application of unstructured preprocessing methods may lead to critical errors such as removal of essential spectral peaks. Denoising methods improve prediction accuracy by reducing high-frequency noise. Scattering correction techniques minimize the effects of particle size and light path variation. Baseline correction methods, including first and second derivatives, and CR, effectively eliminate baseline drift and enhance feature resolution. Scaling methods adjust the relative contribution of variables, although improper application may amplify noise. While satisfactory prediction can be achieved using theory-based wavelength selection for downscaling, data-based selection through statistical methods enables efficient identification of informative wavelengths, reducing data dimensionality. In both procedures, the most effective approach is to apply optimal strategies by comparing each method based on data characteristics. Although prediction models developed under laboratory conditions often show high performance, their application to field conditions requires further validation. Recent studies have demonstrated that prediction models can be applied to on-site measurement through model validation using performance metrics, spiking with field samples, and using soil spectral libraries. Several technological and methodological developments further strengthen the applicability of Vis-NIR spectroscopy. Ensemble modeling and transfer learning have been investigated to improve model generalizability and robustness, particularly across diverse soil types and environmental conditions. Advancements in sensor miniaturization and IoT technologies facilitate the deployment of Vis-NIR sensors on autonomous platforms to allow high-resolution and real-time monitoring. Integrating remote sensing enables multiscale soil assessment by linking localized ground data with broader spatial observations. Vis-NIR spectroscopy combined with machine learning algorithms serves as a valuable strategy for real-time and site-specific soil nutrient prediction. Therefore, this approach will support the implementation of precision nutrient management, contributing to more efficient fertilizer use and the sustainability of agricultural systems. This review integrates and discusses the critical factors influencing prediction accuracy, including data preprocessing strategies, algorithm selection, and environmental variability. While recent developments such as transfer learning and ensemble modeling show potential, further efforts are needed to improve model generalizability and ensure practical implementation under diverse agricultural conditions.

## Figures and Tables

**Table 1:** Inclusion and exclusion criteria for literature screening.

Inclusion Criteria	Exclusion Criteria
Studies focusing on soil, soil moisture, soil organic and inorganic carbon, and soil nutrients	Studies focused on heavy metals, microplastics, or soil contaminants
Studies specifically focusing on Vis-NIR spectral data	Studies primarily analyzing vegetation indices or plant physiological indicators
Application of machine learning or deep learning	Studies not using NIR or Vis-NIR spectroscopy
Studies aiming to predict soil properties using spectral data	Studies without predictive modeling or soil-based focus

**Table 2:** Theoretical absorption wavelength range of molecular bonds.

Bond or Chemical Substances	Wavelength (nm)	Reference
C–H (sp^3^)	3333–3571	[85]
C–H (sp^2^)	3226–3333	
C–H (sp)	3030	
C≡C, C≡N	4348–4545	
O–H, N–H	2703–3333	
C=O	5495–6098	
C–O	7937–9523	
Carbonate	2335, 3968, 6993	[11]
Organic matter	1100, 1600, 1700, 1800, 2000, 2200–2400, 3413–3509, 5,14, 5952, 6135, 6250–6369, 6535, 7143	
Water	1400, 1900	

**Table 4:** Summary of machine learning algorithms applied to predict soil properties using Vis-NIR.

Algorithm	Characteristics	Limitations	References
Partial least square regression (PLSR)	Effective for reducing collinearity	Poor for nonlinear relationships	[7,92]
Support vector machine regression (SVMR)	Handles nonlinear relationships with high generalization performance	Less effective for overlapping data	[15,93]
Random forest (RF)	Robust to overfitting and noise	Decreased accuracy on complex dataset	[94,95]
Gradient boosting regression trees (GBRTs)	High prediction accuracy through gradient-based optimization	Weakness in handling noise	[15,96]
Extreme gradient boost (XGBoost)	Improves generalization and prevents overfitting	Complex hyperparameter tuning	[97]
Extreme learning machine (ELM)	Overcomes slow learning and high generalization performance	Susceptible to overfitting due to lack of structural risk control	[98]
Cubist	Handles nonlinear relationships with intricate datasets	limited interpretability due to rule-based structure	[24,99]
Artificial neural network (ANN)	Supported by advanced mathematical models and software tools	Require integration with optimization algorithm	[100]
Convolutional neural network (CNN)	Improves the generalization of the network and prevent overfitting	Complex architecture tuning	[101]
Backpropagation neural network (BPNN)	Learns through error backpropagation; effective for nonlinear relationships	Slow training rate and risk of convergence to local minima	[43,101]

**Table 5:** Summary of studies on soil water content prediction using Vis-NIR spectroscopy.

Study Environment	Soil Water Type	Soil Water Content (%)	Spectral Data Source	Preprocessing Method	Prediction Method	Prediction Performance	Reference
Field	At a depth of 15 cm	19.7	UAV		Bay-ANN	R^2^ = 0.85, RMSE = 1.1	[118]
Laboratory	Soil-water content at pF 3	16 ± 6	Labspec5100 spectrometer	SG, PCA, gap-segmented second-derivative	PLSR	R^2^ = 0.79–0.84, RMSE = 2.2–2.9, RPIQ = 1.7–2.3	[116]
Laboratory	Available water capacity	14.5 ± 6	Labspec^®^ vis–NIR spectrometer	CR, SG, 1D	Cubist	R^2^ = 0.70, RMSE = 3.3	[106]
Laboratory	Field capacity	39.6 ± 11.0	ASD FieldSpec^®^3 spectroradiometer	SG, 1D, PCA	PLSR, PLS-SVM	R^2^ = 0.70, RMSE = 6.68, RPD = 1.81	[119]
	Permanent wilting point	20.1 ± 10.3				R^2^ = 0.78, RMSE = 4.41, RPD = 2.12	
Laboratory and field	Soil water content	12.6 ± 8.4	Corona fiber VISNIR spectrophotometer	SG, 1D, max normalization	PLSR	Laboratory R^2^ = 0.98, RMSE = 1.65, RPD = 5.12 Field R^2^ = 0.75, RMSE = 2.50, RPD = 3.38	[120]
Laboratory	Soil water content	0.18 ± 0.04	ASD FieldSpec^®^4 spectroradiometer	SG, SNV, MSC, 1D, 2D, Log T, normalization	PLSR	log(1/R) + SG + SNV + 1D R^2^ = 0.80, RMSE = 0.01, RPD = 2.09	[67]

1D, first-order derivative; 2D, second-order derivative; Bay-ANN, Bayesian artificial neural network; CR, continuum removal; Log T, logarithmic transformation; MSC, multiplicative scatter correction; PCA, principal component analysis; PLS, partial least squares; PLSR, partial least-squares regression; SG, Savitzky–Golay filter; SNV, standard normal variate; SVM, support vector machine; UAV, unmanned aerial vehicle.

**Table 6:** Summary of studies on soil organic and inorganic carbon content prediction using Vis-NIR spectroscopy.

Study Environment	Nutrient	Soil Nutrient Content (%)	Spectral Data Source	Preprocessing Method	Prediction Method	Prediction Performance	Reference
Field	SOM	1.82 ± 0.26	Fieldspec^®^ ProFR spectrometer, GF-1 satellite data	SG, PCA, standardization	PLSR, RF	RF RMSE = 0.18, RPIQ = 1.99	[95]
Laboratory	SOC	1.52 ± 0.02	FT-NIR probe	Continuous wavelets transform	Cubist	R^2^ = 0.44, RMSE = 0.31, RPD = 1.32	[9]
	SIC	0.34 ± 0.02				R^2^ = 0.42, RMSE = 0.24, RPD = 1.36	
Laboratory	SOC	1.60 ± 0.49	Fiber-type vis-NIR spectrophotometer	SG, 1D, max normalization	RF	R^2^ = 0.84, RMSE = 0.14, RPD = 2.55	[124]
Field	SOC	1.60 ± 0.49				R^2^ = 0.75, RMSE = 0.17, RPD = 2.04	
Laboratory	SOC	0.40 ± 0.65	ASD FieldSpec^®^3 spectroradiometer	SG, 1D	RF, RF-SVM, ACO-iPLS	RF-SVM R^2^ = 0.91, RMSE = 0.27, RPD = 2.41	[123]
Laboratory	SIC	1.83 ± 0.46	ASD FieldSpec^®^4 spectroradiometer	CARS, PSO, ACO, IRF, IRIV	1-CNN, 2-CNN, LSTM, DBN	IRF-1-CNN R^2^ = 0.90, RMSE = 0.15, RPIQ = 4.20	[104]
Laboratory	SOC	0.20 ± 0.05	ASD FieldSpec^®^5 spectroradiometer	SG, 1D, 2D, MSC, SNV	PLSR	SG-1D R^2^ = 0.66, RMSE = 0.03, RPIQ = 2.19	[122]
	SIC	33.1 ± 28.3				SG-1D R^2^ = 0.93, RMSE = 0.26, RPIQ = 5.08	
Laboratory	SOM	4.17 ± 2.04	PSR-1100F portable ground-object spectrometer	SG, 1D, CR, reciprocal, logarithmic, first derivative of reciprocal, and first derivative of logarithmic	PLS, RF, SVM, XGBoost, BPNN	PLSR-FDR RPD = 1.458, RPIQ = 1.488	[43]
Laboratory	SOC	0.48 ± 0.26	ASD FieldSpec^®^3 spectrometer	SG, 1D, 2D, PCA, SNV	PLSR, SVMR	SVMR-1D R^2^ = 0.84, RMSE = 0.12, RPD = 2.47	[7]
Laboratory	SOM	1.84 ± 0.36	ASD FieldSpec^®^4 spectroradiometer	SG, SNV, MSC, 1D, 2D, Log T, normalization	PLSR	SG + MSC + 1D R^2^ = 0.98, RMSE = 0.45, RPD = 8.56	[67]
Laboratory	SOC (0–10 cm)	0.92–1.6	ASD FieldSpec^®^3 spectrometer	SG, Log T, PCA	PLSR, SVM	SVM R^2^ = 0.87, RMSE = 0.13, RPD = 2.8	[103]
	SOC (10–40 cm)	0.7–1.3				SVM R^2^ = 0.93, RMSE = 0.35, RPD = 2.5	
Laboratory	SOC	2.50 ± 7.42	ASD LabSpec 2500	SG, 1D, 2D, SNV, Log T, CR	PLSR-Log T, RF-SC-1D, Cubist-CR, MARS-SG-1D	RF-SG-1D R^2^ = 0.94, RMSE = 1.78	[77]

1-CNN, one-dimensional convolutional neural network; 1D, first-order derivative; 2-CNN, two-dimensional convolutional neural network; 2D, second-order derivative; ACO, ant colony optimization; ACO-iPLS, ant colony optimization–interval partial least squares; BPNN, backpropagation neural network; CARS, competitive adaptive reweighted sampling; CNN, convolutional neural network; DBN, deep belief network; IRF, interval random frog; IRIV, iteratively retaining informative variables; Log T, logarithmic transformation; LSTM, long short-term memory network; MARS, multivariable adaptive regression splines; MSC, multiplicative scatter correction; PCA, principal component analysis; PLS, partial least squares; PLSR, partial least-squares regression; PSO, particle swarm optimization; RF, random forest; SG, Savitzky–Golay filter; SIC, soil inorganic carbon; SNV, standard normal variate; SOC, soil organic carbon; SOM, soil organic matter; SVM, support vector machine; SVMR, support vector machine regression; XGBoost, extreme gradient boost.

**Table 7:** Summary of studies on soil nutrient content prediction using Vis-NIR spectroscopy.

Study Environment	Nutrient	Soil Nutrient Content (mg kg^−1^)	Spectral Data Source	Preprocessing Method	Prediction Method	Prediction Performance	Reference
Field	TN	1340 ± 140	Fieldspec^®^ ProFR spectrometer GF-1 satellite data	SG, PCA, standardization	PLSR, RF	PLSR RMSE = 110, RPIQ = 1.59	[95]
Laboratory	TN	1100 ± 400	ASD FieldSpec^®^3 spectrometer	SG, 1D, CR	PLSR, SVMR, BPNN, ELM,	ELM R^2^ = 0.65, RMSE = 200	[18]
Laboratory	AN	24.6 ± 16.7	ASD FieldSpec^®^3 spectrometer	SG, 1D, 2D, PCA, SNV	PLSR, SVMR	PLSR-1D R^2^ = 0.49, RMSE = 12.34, RPD = 1.40	[7]
	AP	123.5 ± 90.7				PLSR-1D R^2^ = 0.71, RMSE = 45.75, RPD = 1.83	
	AK	88.2 ± 52.0				PLSR-1D R^2^ = 0.70, RMSE = 34.02, RPD = 1.82	
Laboratory	TN	0.08 ± 0.03	NIRs-XDS	Wavelet function (autoscale, GLSW, detrend + GLSW, EPO)	SVMR, PLS-ANN, GBRT	GBRT-EPO r = 0.925, RMSE = 0.013, RPD = 2.6349	[15]
	TP	6.38 ± 14.0				GBRT-EPO r = 0.967, RMSE = 4.825, RPD = 3.9229	
	TK	0.33 ± 0.30				GBRT-EPO r = 0.908, RMSE = 0.126, RPD = 2.3805	
Laboratory	Oxalate-extractable P	220.9 ± 290.0	ASD FieldSpec^®^4 spectrometer	SG, SNV	PLSR, RF, 1-CNN	1-CNN R^2^ = 0.88, RMSE = 101.2 RPIQ = 2.49	[105]
Laboratory	TN	1350 ± 160	ASD FieldSpec^®^4 spectrometer	SG, SNV, MSC, 1D, 2D, Log T, normalization	PLSR	R + SG + NOR + 2D R^2^ = 0.98, RMSE = 20, RPD = 6.67	[67]
	Nitrate	4.68 ± 2.82				log(1/R) + SG + 1D R^2^ = 0.90, RMSE = 0.62, RPD = 3.07	
	AP	26.0 ± 17.2				log(1/R) + SG + MSC + 2D R^2^ = 0.85, RMSE = 5.75, RPD = 3.58	
	AK	142.6 ± 56.9				R + SG + SNV + 2D R^2^ = 0.89, RMSE = 1.39, RPD = 2.91	
Laboratory	Topsoil TN (0–10 cm)	5000–8000	ASD FieldSpec^®^3 spectrometer	SG, Log T, PCA	PLSR, SVM	SVM R^2^ = 0.91, RMSE = 1000, RPD = 2.4	[103]
	Subsoil TN (10~40 cm)	2000–4000				SVM R^2^ = 0.89, RMSE = 2600, RPD = 2.4	
Laboratory	TN	1600 ± 3500	ASD LabSpec 2500	SG, 1D, 2D, SNV, Log T, CR	PLSR, RF, Cubist, MARS	Cubist-CR R^2^ = 0.92, RMSE = 1000	[77]

1-CNN, one-dimensional convolutional neural network; 1D, first-order derivative; 2D, second-order derivative; AK, available potassium; AN, available nitrogen; ANN, artificial neural network; AP, available phosphorus; BPNN, backpropagation neural network; CNN, convolutional neural network; CR, continuum removal; ELM, extreme learning machine; EPO, external parameter orthogonalization; GLSW, generalized least squares weighting; Log T, logarithmic transformation; MARS, multivariable adaptive regression splines; MSC, multiplicative scatter correction; PCA, principal component analysis; PLS, partial least squares; PLSR, partial least-squares regression; RF, random forest; SG, Savitzky–Golay filter; SNV, standard normal variate; SVM, support vector machine; SVMR, support vector machine regression; TN, total nitrogen; TK, total potassium; TP, total phosphorus.

## Data Availability

No new data were created or analyzed in this study. Data sharing is not applicable to this article.

## Abbreviations

The following abbreviations are used in this manuscript:

1D	First-order derivative
2D	Second-order derivative
AK	Available potassium
ANN	Artificial neural network
AP	Available phosphorus
BPNN	Backpropagation neural network
CARS	Competitive adaptive reweighted sampling
CNN	Convolutional neural network
CR	Continuum removal
ELM	Extreme learning machine
GBRT	Gradient boosting regression trees
MSC	Multiplicative scatter correction
NOR	Normalization
PCA	Principal component analysis
PLSR	Partial least-squares regression
R^2^	Coefficient of determination
RMSE	Root mean square error
RPD	Residual predictive deviation
RPIQ	Ratio of performance to interquartile distance
SG	Savitzky–Golay filter
SIC	Soil inorganic carbon
SNV	Standard normal variate
SOC	Soil organic carbon
SVM	Support vector machine
SVMR	Support vector machine regression
TN	Total nitrogen
Vis-NIR	Visible and near-infrared
XGBoost	Extreme gradient boost
